# Can Calcaneofibular Ligament Repair Correct Hindfoot Varus Alignment in the Management of Chronic Lateral Ankle Instability?

**DOI:** 10.7759/cureus.82768

**Published:** 2025-04-22

**Authors:** Kensei Yoshimoto, Mitsuki Kumaki, Takumi Koseki, Ayako Tominaga, Masahiko Noguchi, Ken Okazaki

**Affiliations:** 1 Orthopaedic Foot and Ankle Center, Shiseikai Daini hospital, Tokyo, JPN; 2 Orthopaedic Foot and Ankle Center, Shiseikai Daini Hospital, Tokyo, JPN; 3 Department of Orthopedics, Tokyo Women’s Medical University, Tokyo, JPN

**Keywords:** alignment, ankle instability, anterior talofibular ligament, arthroscopic repair, calcaneofibular ligament, chronic lateral ankle instability, hindfoot, lateral ankle ligament instability

## Abstract

Background: Hindfoot varus alignment is a risk factor for chronic lateral ankle instability (CLAI) and recurrent ankle instability following anterior talofibular ligament (ATFL) repair for CLAI. This study aimed to assess whether combining calcaneofibular ligament (CFL) repair with ATFL repair can correct hindfoot varus alignment and reduce recurrent ankle instability.

Methods: This study retrospectively examined 74 ankles from 71 patients with hindfoot varus alignment who had lateral ankle ligament repair for CLAI. Arthroscopic ATFL repair was performed on 37 ankles (from 36 patients) between 2018 and 2021 (ATFL-only group), whereas open ATFL and CFL repair were conducted on another 37 ankles (from 35 patients) between 2021 and 2023 (CFL-repair group). Long axial hindfoot alignment radiographs were utilized to measure tibiocalcaneal angles (TCA) pre- and postoperatively. The primary outcome includes recurrent ankle instability (i.e., respraining of the operated ankle after surgery), whereas the secondary outcome involves Self-Administered Foot Evaluation Questionnaire (SAFE-Q) scores.

Results: A comparison of patient demographics between the two groups revealed no marked differences. Additionally, pre- and postoperative TCA demonstrated that neither ATFL repair only nor CFL repair combined with ATFL repair corrected hindfoot varus alignment. Furthermore, no significant differences were observed in postoperative recurrent ankle instability and SAFE-Q scores between the two groups.

Conclusion: Combining ATFL and CFL repair could not correct hindfoot varus alignment, and this procedure for CLAI with hindfoot varus alignment was not more effective in reducing recurrent ankle instability than ATFL repair alone.

## Introduction

Ankle sprain is a predominant injury that usually heals with conservative treatment. However, approximately 20% of patients experience chronic lateral ankle instability (CLAI)[1]. Repetitive ankle sprain and chronic instability cause degenerative changes if not adequately treated [2]. Surgical intervention is indicated for patients with mechanical instability after nonoperative modality failure.

Only anterior talofibular ligament (ATFL) repair with open or arthroscopic modified Broström procedure for CLAI is a widely accepted procedure with good clinical outcomes [3,4]. However, hindfoot varus alignment is a popular risk factor for CLAI [5] and recurrent ankle instability after ATFL repair [6]. Furthermore, this malalignment results in poor clinical outcomes after arthroscopic ATFL repair [6]. Therefore, hindfoot varus alignment correction is crucial for CLAI [7]. Calcaneus osteotomy is a reliable procedure for hindfoot varus alignment correction [7], but it is more invasive than ligament repair. Conversely, calcaneofibular ligament (CFL) injury could induce subtalar instability [8], causing hindfoot varus alignment. Therefore, CFL repair is envisaged to correct the hindfoot varus alignment. However, no study assessed the effectiveness of CFL repair in hindfoot varus alignment correction and postoperative recurrent ankle instability reduction.

This study aimed to assess whether combining ATFL repair with CFL repair could achieve better correction of hindfoot varus alignment and postoperative ankle instability reduction compared to ATFL repair alone. It was considered valuable to compare these two surgical procedures because a recent study suggested that repairing only the ATFL without addressing the injured CFL results in persistent ankle instability [9]. Further, this study hypothesized that both ATFL and CFL repair could correct hindfoot varus alignment as well as calcaneus osteotomy and reduce postoperative ankle instability.

## Materials and methods

Study population and study design

**Figure 1:**
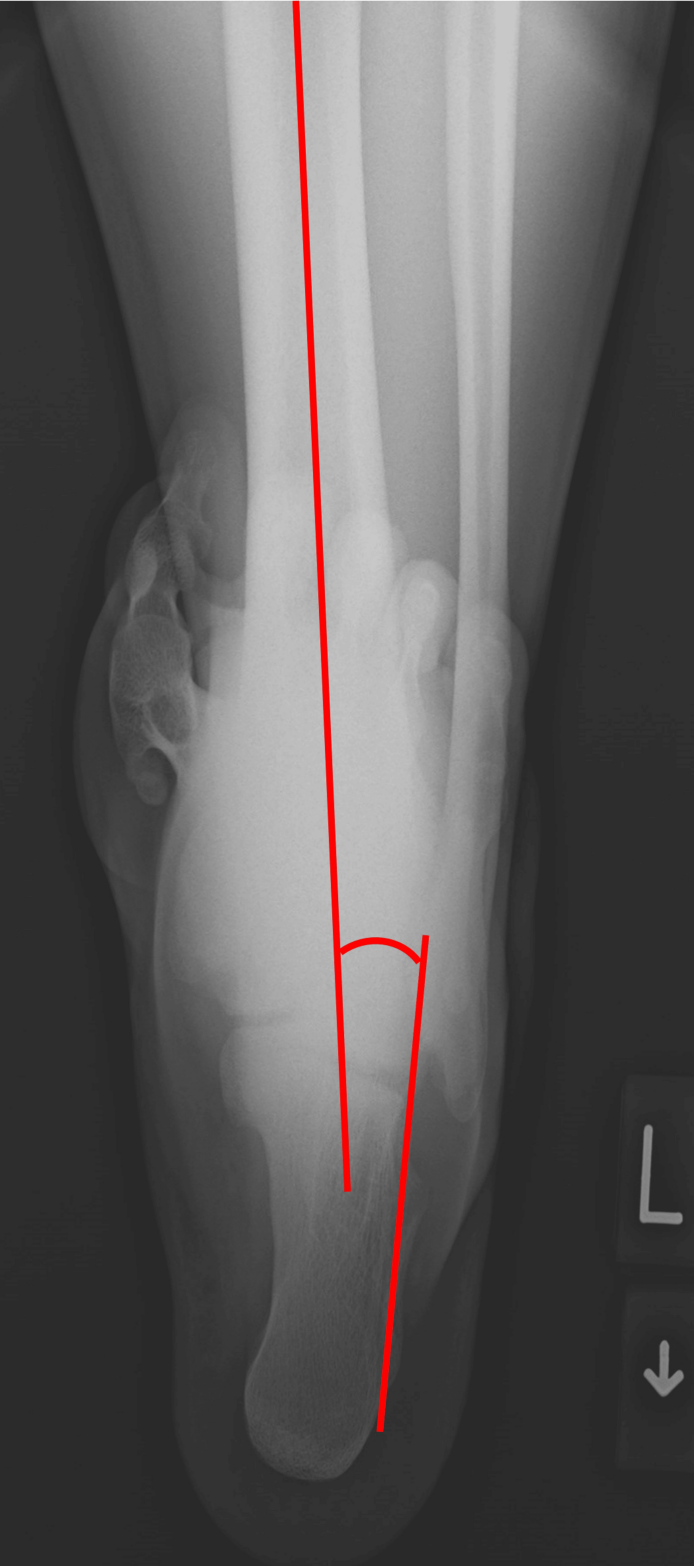
The tibiocalcaneal angle is the angle between the tibial axis and the calcaneal axis (defined as a line parallel to the lateral calcaneal wall).

Patient records from 2018 to 2023 were analyzed, during which lateral ankle ligament repair for CLAI was performed on 245 ankles from 235 patients. Of this number, the retrospective study screened 108 ankles from 103 patients who exhibited hindfoot varus alignment. Hindfoot varus alignment is defined as a preoperative tibiocalcaneal angle (TCA) of 2.7 degrees or more (Figure 1), measured on long axial hindfoot alignment radiographs [10], which serves as the cut-off value for recurrent ankle instability after arthroscopic ATFL repair for CLAI [11]. TCA is the most commonly used measurement for hindfoot alignment [12]. The indication for surgery included persistent pain that did not respond to conservative treatment and signs of ankle instability on physical examination [13]. The physical examination for the ankle instability depends on assessing anterior drawer and talar tilt laxity, which were considered pathological when a “nonstop” was observed [13,14].

This study excluded 34 patients (32 ankles) because of a short (<1 year) follow-up period (18 ankles from 17 patients), ankle osteoarthritis, stage 2 or more following the Takakura classification [15] (nine ankles from eight patients), foot and ankle surgery history (five ankles from five patients), and neuromuscular disorders (two ankles from two patients).

Ultimately, this study included 74 ankles from 71 patients. Of this number, arthroscopic ATFL repair was performed on 37 ankles from 36 patients between 2018 and 2021 (ATFL-only group), whereas open repairs for both the ATFL and CFL were conducted on 37 ankles from 35 patients between 2021 and 2023 (CFL-repair group), due to the high rate of recurrent ankle instability associated with arthroscopic ATFL repair in cases of hindfoot varus alignment [6].

Surgical procedure

**Figure 2:**
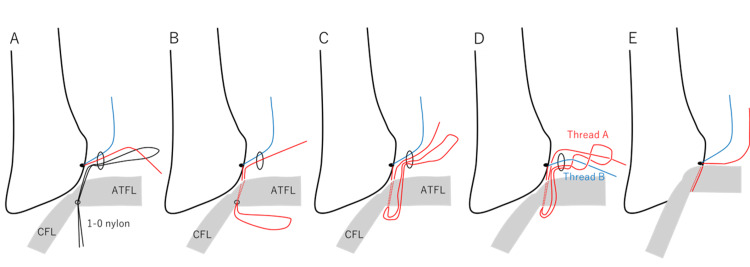
Details of arthroscopic ATFL repair An 18-G hollow needle with a 1–0 nylon thread was inserted through the fibula tip to penetrate the inferior bundle of the anterior talofibular ligament after placing a suture anchor at the inferior edge of the anteroinferior tibiofibular ligament. The nylon loop was retrieved via the accessory anterolateral portal. One limb of the suture anchor was then passed through the nylon loop (A), and the opposite end of the nylon loop was pulled out percutaneously (B). Subcutaneous passage of the suture loop through the accessory anterolateral portal was facilitated (C). The loop was twisted to lead thread B, and the loop was then twisted again to lead thread A (D). Thread A was pulled lightly while thread B was pulled tightly to reattach the footprint of the lateral ankle ligament. Finally, a square knot and a granny knot were made twice with a knot pusher (E). ATFL: anterior talofibular ligament

A single orthopedic surgeon performed all the surgeries. The surgeon conducted an arthroscopic examination using a 2.7-mm, 30-degree arthroscope. The medial midline portal was used for a viewing portal [16], and the accessory anterolateral (acAL) portal was used for a working portal. Arthroscopic ATFL repair was performed following the previously described procedure [11]. In brief, a suture anchor was placed at the inferior edge of the anteroinferior tibiofibular ligament (AITFL) via the acAL portal [17]. One limb of the suture anchor penetrated the inferior bundle of the ATFL. The ATFL was then reattached to the footprint using the modified lasso loop stitch technique (Figure 2)[16].

**Figure 3:**
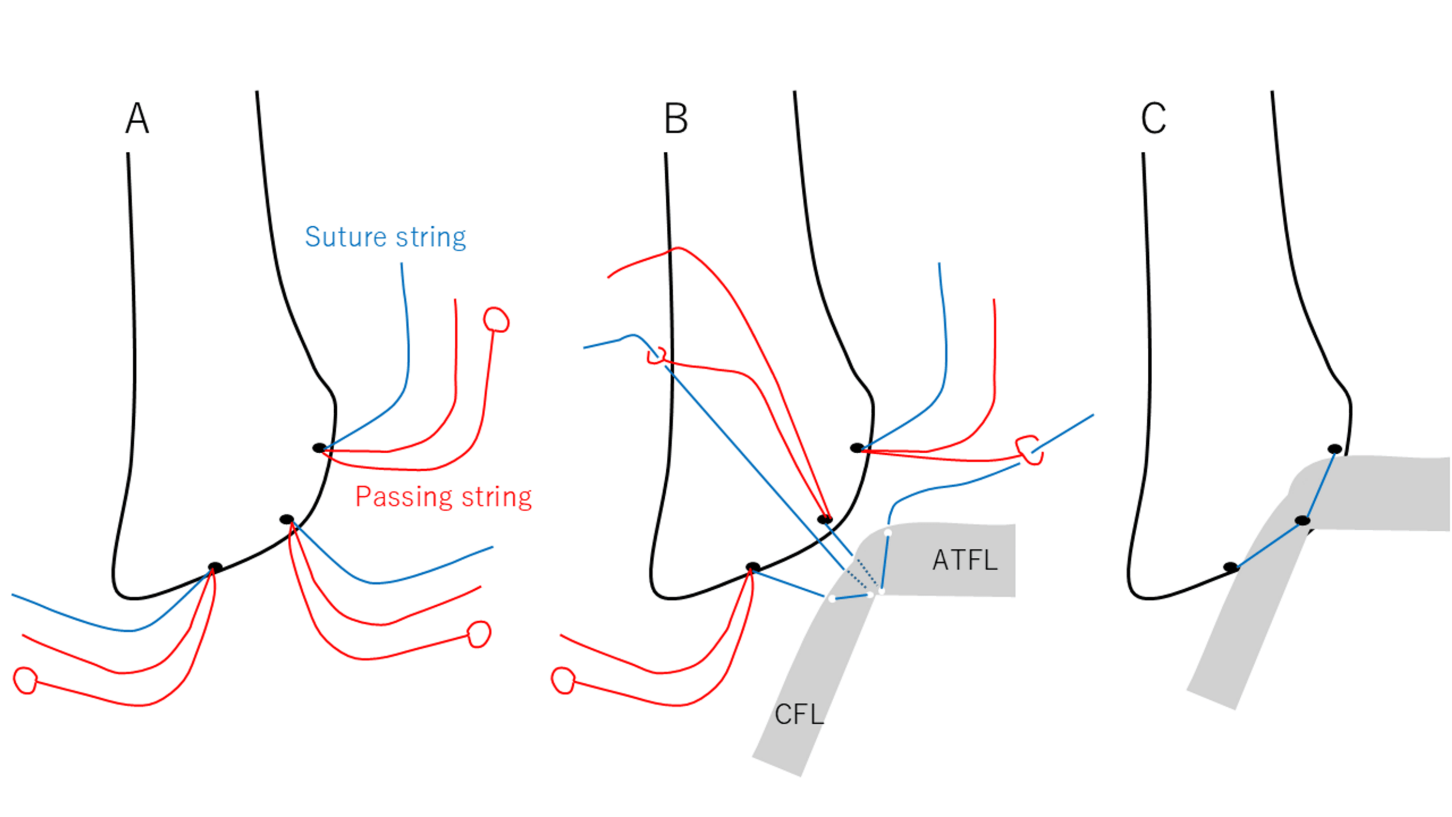
Details of open ATFL and CFL repair DEX Knotless FiberTak® suture anchor (Arthrex, Naples, FL, USA) was inserted into the superior edge of the ATFL attachment, OT, and the posterior edge of the CFL attachment (A). A suture string that was inserted in the posterior edge of the CFL attachment was threaded CFL and capsule complex and passed into the small circle of a passing string inserted in OT. A suture string that was inserted in the OT was threaded ATFL and capsule complex and passed into the small circle of a passing string inserted in the superior edge of the ATFL attachment (B). The other passing string was drawn to attach the ATFL and CFL to the lateral malleolus (Figure 3C). ATFL: anterior talofibular ligament; CFL : calcaneofibular ligament; OT: obscure tubercle

Open repair of both the ATFL and CFL was performed with a 2 cm-long skin incision along the anterior border of the fibula and centered on the obscure tubercle (OT)[17]. The capsule-ligament complex was incised from just distal to the AITFL attachment to the posterior edge of the CFL, along the anterior border of the lateral malleolus without capsule and ligament separation [18]. DEX Knotless FiberTak® suture anchor (Arthrex, Naples, FL, USA) was then inserted into the superior edge of the ATFL attachment, OT, and the posterior edge of the CFL attachment (Figure 3A). This suture anchor consisted of one suture string and two passing strings, which enabled ligament repair without knots. A suture string that was inserted in the posterior edge of the CFL attachment was threaded CFL and capsule complex and passed into the small circle of a passing string inserted in OT. Afterward, a suture string that was inserted in the OT was threaded ATFL and capsule complex and passed into the small circle of a passing string inserted in the superior edge of the ATFL attachment (Figure 3B). The other passing string was drawn to attach the ATFL and CFL to the lateral malleolus (Figure 3C).

All patients were allowed full weight-bearing from day one postoperatively with cast immobilization (until 2022) or ankle brace (from 2023). Because the maximum stress force on the ATFL is only 11.3 N during the stance phase of gait, rigid cast immobilization was considered unnecessary [19]. The cast was removed for one week postoperatively. An ankle brace was used for five weeks after surgery. The patients performed exercises with a physical therapist after wearing the ankle brace to restore muscle strength and motion range. They were also allowed to resume full sports activities once they demonstrated normal ankle function during sport-specific drills at least 5 weeks after surgery.

Evaluation

Long axial hindfoot alignment radiographs were taken preoperatively and 1 year postoperatively [10]. The TCA, which is the angle between the tibial and calcaneal axes (defined as a line parallel to the lateral calcaneal wall), was measured (Figure 1) [12,20]. The adequate reliability of this measurement has already been demonstrated [6]. Positive TCA values indicate hindfoot varus. Furthermore, the flexibility of hindfoot varus alignment was evaluated by two orthopedic surgeons using the Coleman block test, and the interobserver reproducibility of this test was assessed.

The primary outcome includes recurrent ankle instability. During their latest visit, patients were confirmed if their operated ankle had been resprained postoperatively. Patients who experienced resprain of their operated ankle were diagnosed with recurrent ankle instability [6,11,13]. The Self-Administered Foot Evaluation Questionnaire (SAFE-Q) was used to assess secondary outcomes preoperatively and at the latest visit [21,22].

Statistical analysis

Statistical analysis was conducted with JMP Pro version 17.0 software (SAS Institute, Cary, NC, USA). Differences between the two groups were assessed with Fisher’s exact probability test and the Mann-Whitney U test for categorical and continuous variables, respectively. P-values of less than 0.05 indicated statistical significance.

## Results

There were no significant differences in patients’ demographics and preoperative SAFE-Q scores between the two groups (Table 1).

**Table 1:** Comparison of patient demographics between the two groups ATFL: anterior talofibular ligament; CFL: calcaneofibular ligament; BMI: body mass index; TCA: tibiocalcaneal angle; SAFE-Q: The Self-Administered Foot Evaluation Questionnaire Values are mean ± standard deviation

	ATFL-only (37 ankles)	CFL repair (37 ankles)	P-value
Age (years)	38.1 ± 17.1	37.5 ± 15.3	0.867
Males	17 (45.9%)	16 (43.2%)	
Females	20 (54.1%)	21 (56.8%)	0.815
BMI (kg/m^2^)	23.3 ± 4.0	23.9 ± 3.2	0.163
Follow-up periods (months)	22.8 ± 12.8	18.8 ± 8.2	0.201
Sports participation	21 (56.8%)	17 (45.9%)	0.283
Preoperative SAFE-Q			
Pain related	66.5 ± 23.5	59.0 ± 25.8	0.249
Physical functioning	82.5 ± 19.0	81.2 ± 16.5	0.436
Social functioning	77.7 ± 26.5	71.0 ± 25.7	0.214
Shoe related	85.4 ± 18.4	76.7 ± 26.3	0.189
General health	75.6 ± 20.5	69.4 ± 24.8	0.324

The mean pre- and postoperative TCAs were 5.8 ± 2.8 degrees (95% confidence interval (CI), 4.8 to 6.7) and 5.6 ± 3.0 degrees (95% CI, 4.6 to 6.6) in ATFL-only group and 5.7 ± 2.9 degrees (95% CI, 4.7 to 6.6) and 5.6 ± 3.0 degrees (95% CI, 4.6 to 6.6) in the CFL-repair group, respectively. The 95% CI for the differences between pre- and postoperative TCA was −0.7° to 0.4° in the ATFL-only group and −0.5° to 0.4° in the CFL-repair group, which were similar to the measurement error when the same examiner measured TCA twice (−0.5°−0.4°) (Table 2). The interobserver reproducibility of hindfoot flexibility assessment using the Coleman block test was limited to 0.29.

**Table 2:** Pre- and postoperative TCA TCA: tibiocalcaneal angles; ATFL: anterior talofibular ligament; CFL: calcaneofibular ligament; CI: confidence interval

	ATFL-only (37 ankles)	CFL repair (37 ankles)	P-value
Preoperative TCA (degrees)	5.8 ± 2.8 (95% CI: 4.8 to 6.7)	5.7 ± 2.9 (95% CI, 4.7 to 6.6)	0.622
Postoperative TCA (degrees)	5.6 ± 3.0 (95% CI: 4.6 to 6.6)	5.6 ± 3.0 (95% CI, 4.6 to 6.6)	0.869
95% CI of differences	-0.7 to 0.4	-0.5 to 0.4	

Further, no marked differences were found in postoperative recurrent ankle instability and SAFE-Q scores between the two groups. Two patients in the ATFL-only group underwent revision surgery for recurrent ankle instability, and no patients in the CFL-repair group underwent revision surgery. Wound infection occurred in one patient in the ATFL-only group and two patients in the CFL-repair group. Other surgical complications, such as nerve injury, vascular injury, and tendon injury, did not occur (Table 3).

**Table 3:** Comparison of postoperative outcomes between the two groups ATFL: anterior talofibular ligament; CFL: calcaneofibular ligament; SAFE-Q: The self-administered foot evaluation questionnaire Values are mean ± standard deviation. *P <0.05

	ATFL-only (37 ankles)	CFL repair (37 ankles)	P-value
Recurrent instability (ankles)	13 (35.1%)	11 (29.7%)	0.619
Revision surgery (ankles)	2 (0.5%)	0 (0%)	0.493
Complications (ankles)	1 (0.3%)	2 (0.5%)	1
Postoperative SAFE-Q			
Pain related	88.8 ± 16.8	90.4 ± 13.4	0.788
Physical functioning	95.0 ± 9.9	96.3 ± 5.6	0.980
Social functioning	96.4 ± 9.0	97.1 ± 4.9	0.423
Shoe related	93.2 ± 12.7	88.7 ± 15.6	0.283
General health	94.0 ± 13.9	93.7 ± 8.5	0.139

## Discussion

The overarching finding in this study was that combining open repair of both the ATFL and CFL did not achieve better correction of the hindfoot varus alignment or a lower rate of recurrent ankle instability compared to ATFL-only repair. To the best of our knowledge, this is the first study that assessed whether repair of both the ATFL and CFL for CLAI could correct hindfoot varus alignment.

Hindfoot varus alignment increases the ankle varus moment during weight-bearing, which promotes stress on the lateral ankle ligament [5]. In particular, CFL injury causes subtalar instability [8] and may induce hindfoot varus alignment. Therefore, it was hypothesized that CFL repair corrects hindfoot varus alignment; however, this hypothesis was not supported. Several reasons may explain why CFL repair could not correct hindfoot varus alignment in this study. First, the effect of the interosseous talocalcaneal and cervical ligaments that work together to stabilize the subtalar joint [23,24] was not considered. Second, CFL repair could not correct rigid hindfoot varus alignment. However, the flexibility of hindfoot varus alignment in this study was unclear. The interobserver reproducibility of hindfoot flexibility assessment using the Coleman block test was limited to 0.29 in this study, likely because the patients had slight hindfoot varus, making accurate evaluation difficult. Third, hindfoot varus deformity was not solely due to ligament-related instability but also resulted from various bone and joint deformities in the foot and ankle.

Long-term follow-up studies of ATFL repair without CFL repair demonstrated good clinical outcomes [25], but an increasing number of articles have focused on the importance of CFL. A biomechanical study observed persistent ankle instability only when the ATFL was repaired without addressing the injured CFL [9], and only ATFL repair without addressing the injured CFL did not correct a normal strain pattern of the CFL [26]. Furthermore, repairing only the ATFL while neglecting a CFL injury may cause recurrent instability [27]. However, the present study could not demonstrate the effectiveness of CFL repair for hindfoot varus alignment. This result was attributed to the residual hindfoot varus alignment after CFL repair.

Hindfoot varus alignment is a popular risk factor for CLAI [5] and recurrent ankle instability after ATFL repair [6]. CFL repair could not correct hindfoot varus alignment; thus, surgeons should consider calcaneal osteotomy if the surgery is aimed at correcting hindfoot varus alignment [7]. However, this study showed good SAFE-Q scores regardless of residual hindfoot varus alignment. Therefore, calcaneal osteotomy might not be necessary for primary surgery but could be required for revision surgery. Moreover, because this study could not determine the cut-off value of TCA to require calcaneus osteotomy, further investigation was needed.

This research has several limitations. First, stress radiography was not assessed. This procedure was deemed unnecessary to evaluate the laxity of CLAI because of its high false-negative rate [28] and because most surgeons do not commonly depend on it [29]. Second, the condition of the lateral ankle ligaments was not assessed. The evaluation of CFL injuries is particularly important because this study primarily aimed to evaluate the effectiveness of CFL repair. However, accurate diagnostic methods and classification of chronic CFL injuries have not been established [30]. If CFL repair was performed based on an accurate diagnosis of CFL injury, different outcomes might be observed. Third, hindfoot flexibility could not be evaluated, as the patients in this study had slight hindfoot varus, making it very difficult to assess hindfoot flexibility using the Coleman block test. Fourth, this study had a relatively small case series. Additional power may have affected the statistical significance of our outcome variables. Furthermore, this study only recorded short-term outcomes. Longer follow-up periods were necessary to assess long-term functional and degenerative outcomes.

## Conclusions

The effectiveness of combining ATFL and CFL repair for CLAI with hindfoot varus alignment was assessed. Combining ATFL and CFL repair could not correct hindfoot varus alignment, and this procedure for CLAI with hindfoot varus alignment was not more effective in reducing recurrent ankle instability than ATFL repair alone. These results indicated that surgeons should consider calcaneus osteotomy to correct hindfoot varus alignment.
